# Supplementary material for: Association between oral microbiome and seven types of cancers in East Asian population: a two-sample Mendelian randomization analysis
Source: Front Mol Biosci. 2023 Nov 21;10:1327893. doi: 10.3389/fmolb.2023.1327893 (PMC10702768; doi:10.3389/fmolb.2023.1327893)
Supplement: Supplementary file 1 [file DataSheet1.ZIP › Supplementary Materials/MR plots for tongue/tongue═╝/Ovarian cancer/pheno.2879_to_ovarian cancer_leave_one_out.pdf]

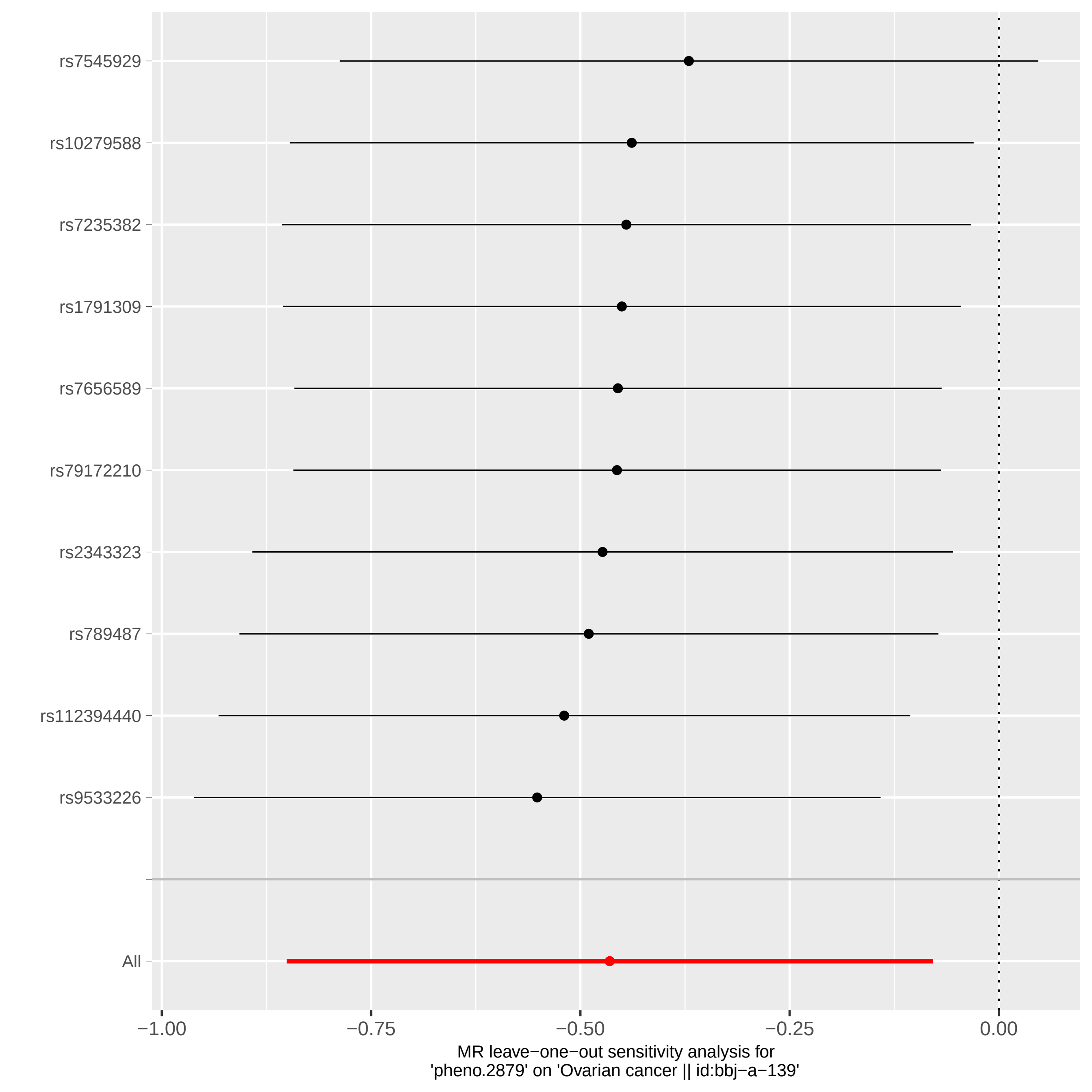

rs7545929
rs10279588
rs7235382
rs1791309
rs7656589
rs79172210
rs2343323
rs789487
rs112394440
rs9533226
All
−1.00
−0.75
−0.50
−0.25
0.00
MR leave−one−out sensitivity analysis for
'pheno.2879' on 'Ovarian cancer || id:bbj−a−139'
